# Correction: Spanish version of the short European Health Literacy Survey Questionnaire HLS-Q12: Transcultural adaptation and psychometric properties

**DOI:** 10.1371/journal.pone.0317154

**Published:** 2025-01-03

**Authors:** Sergio Muñoz-Villaverde, Leticia Serrano-Oviedo, María Martínez-García, Yolanda Pardo, Llüisa Tares-Montserrat, Francisco Javier Gómez-Romero, Paloma Garcimartin

The following information is missing in the Acknowledgments section: We thank the Hospital del Mar Research Institute (IMIM) for its collaboration in carrying out this study.
