# Correction: Spanish version of the short European health literacy survey questionnaire HLS-Q12: Transcultural adaptation and psychometric properties

**DOI:** 10.1371/journal.pone.0334030

**Published:** 2025-10-07

**Authors:** Sergio Muñoz-Villaverde, Leticia Serrano-Oviedo, María Martínez-García, Yolanda Pardo, Llüisa Tares-Montserrat, Francisco Javier Gómez-Romero, Paloma Garcimartin

The corresponding author’s email address is incorrect. Sergio Muñoz-Villaverde email address is: sergiodoctoradoenf@gmail.com
