# Supplementary material for: Spanish version of the short European Health Literacy Survey Questionnaire HLS-Q12: Transcultural adaptation and psychometric properties
Source: PLoS One. 2024 Feb 29;19(2):e0299736. doi: 10.1371/journal.pone.0299736 (PMC10903891; doi:10.1371/journal.pone.0299736)
Supplement: S1 Appendix — (PDF) [file pone.0299736.s001.pdf]

ALFABETIZACIÓN EN SALUD (Cuestionario HLS-Q12).

En una escala de "muy difícil" a "muy fácil", indique cuál es el grado de dificultad que encontraría para realizar las siguientes actividades:

	1. Muy difícil	2. Difícil	3. Fácil	4. Muy fácil
1. encontrar información sobre los tratamientos de enfermedades que le preocupan?	☐	☐	☐	☐
2. entender que debe hacer usted en una emergencia médica?	☐	☐	☐	☐
3. valorar las ventajas e inconvenientes de diferentes opciones de tratamientos?	☐	☐	☐	☐
4. seguir las indicaciones de una medicación?	☐	☐	☐	☐
5. encontrar información sobre como afrontar problemas de salud mental como el estrés y la depresión?	☐	☐	☐	☐
6. entender porque es necesario realizarte pruebas médicas (p.ej. mamografía, azúcar en sangre, presión arterial…)?	☐	☐	☐	☐
7. valorar si la información sobre riesgos para la salud que aparece en los medios de comunicación es fiable (TV, internet u otros)?	☐	☐	☐	☐
8. decidir como puede prevenir enfermedades siguiendo consejos de salud de familiares y amigos?	☐	☐	☐	☐
9. encontrar información sobre actividades saludables como ejercicio, comida sana y nutrición?	☐	☐	☐	☐
10. entender la información que aparece en los envases de alimentos?	☐	☐	☐	☐
11. valorar que actividades del día a día influyen sobre su salud (hábitos alimenticios, ejercicio, etc.)?	☐	☐	☐	☐
12. tomar decisiones para mejorar su salud?	☐	☐	☐	☐
